# Supplementary material for: Phenotypic and Genomic Analysis of Enterobacter ludwigii Strains: Insights into Mechanisms Enhancing Plant Growth Both Under Normal Conditions and in Response to Supplementation with Mineral Fertilizers and Exposure to Stress Factors
Source: Plants (Basel). 2024 Dec 19;13(24):3551. doi: 10.3390/plants13243551 (PMC11677954; doi:10.3390/plants13243551)
Supplement: Supplementary file 1 [file plants-13-03551-s001.zip › Supplementary Table 1,3,4.pdf]

Supplementary Table S1. Morphometric and physiological indices of wheat grown under different microbial fertilizer (MN – mineral nutrition)

	weight leaf, g	weight root, g	weight dry leaf, g	weight dry root, g	height, cm
Water	0.73±0.06	0.87±0.23	0.25±0.03	0.3±0.09	28.67±3.21
MN	1.7±0.09	1.64±0.04	0.63±0.09	0.56±0.07	49.93±2.26
GMG278	1.1±0.17	1.39±0.11	0.39±0.11	0.42±0.02	36.87±3.74
MN-GMG278	2.43±0.3	2.32±0.22	1.1±0.06	0.94±0.03	74.8±2.19
GMG291	0.75±0.03	0.93±0.18	0.25±0.03	0.27±0.05	27.57±1.55
MN-GMG291	1.71±0.14	1.74±0.16	0.59±0.09	0.54±0.04	51.57±1.87
GMG336	0.93±0.1	1.14±0.05	0.34±0.04	0.39±0.01	37.75±2.54
MN-GMG336	1.85±0.05	1.68±0.18	0.67±0.02	0.6±0.03	56.33±0.8
GMG378	0.83±0.14	1.39±0.11	0.26±0.04	0.28±0.06	30.3±1.44
MN-GMG378	1.7±0.05	1.87±0.2	0.64±0.05	0.66±0.05	52.1±0.35

Supplementary Table S3. Morphometric and physiological indicators of plants under stress conditions in experiments. Legend (MF – mineral fertilizer, 278 – microbial fertilizer, NaCl – watering with 1% NaCl solution, PEG – watering with 10% PEG solution).

	weight leaf, g	weight root, g	weight dry leaf, g	weight dry root, g	height, cm	chlorophyll A, mg/g	chlorophyll B, mg/g	carotenoids, mg/g	chlorophyll A/B	chlA+B/car	proline, mg/ml
Water	0.49±0.11	0.45±0.1	0.14±0	0.13±0.03	22.42±2.96	0.29±0.08	0.21±0.08	1.71±0.05	0.26±0.1	2.23±0.09	0.23±0.07
MN	0.8±0.12	0.69±0.13	0.28±0.1	0.2±0.03	27.93±1.29	0.85±0.09	0.46±0.06	1.86±0.07	0.55±0.05	2.39±0.11	0.33±0.04
278	0.74±0.11	0.72±0.06	0.22±0.06	0.19±0.01	28.83±2.95	0.56±0.22	0.32±0.12	1.71±0.02	0.36±0.13	2.45±0.05	0.3±0.1
278-MN	1.15±0.14	0.98±0.09	0.49±0.03	0.33±0.01	41.88±1.21	1.13±0.01	1.11±0.29	1.08±0.33	0.58±0	3.86±0.52	0.54±0.08
PEG	0.31±0.05	0.32±0.08	0.11±0.01	0.16±0.03	21.33±0.44	0.53±0.26	0.27±0.13	1.93±0.07	0.34±0.16	2.36±0.07	0.23±0.06
PEG-MN	0.64±0.08	0.43±0.15	0.22±0.02	0.16±0.02	28.69±2.65	1.16±0.02	1.2±0.23	0.98±0.18	0.58±0.01	4.08±0.48	0.58±0.08
PEG278	0.67±0.08	0.63±0.07	0.18±0.03	0.31±0.05	26.58±3.55	0.47±0.16	0.25±0.08	1.88±0.03	0.3±0.1	2.38±0.03	0.21±0.04
PEG278-MN	0.8±0.11	0.74±0.1	0.32±0.03	0.33±0.03	33.36±2.44	0.97±0.17	0.74±0.26	1.38±0.41	0.58±0.02	2.95±0.66	0.49±0.14
NaCl	0.38±0.06	0.47±0.08	0.14±0.04	0.13±0.02	21.72±1.35	0.54±0.23	0.27±0.11	1.96±0.08	0.35±0.15	2.31±0.08	0.2±0.06
NaCl-MN	0.66±0.13	0.53±0.13	0.23±0.03	0.17±0.02	26.19±1.14	1.12±0.08	0.7±0.07	1.61±0.05	0.59±0	3.07±0.27	0.36±0.03
NaCl278	0.62±0.09	0.5±0.05	0.2±0.05	0.18±0.02	26.67±0.88	0.83±0.07	0.44±0.06	1.89±0.08	0.5±0.03	2.55±0.1	0.28±0.05
NaCl278-MN	0.89±0.24	0.79±0.09	0.37±0.09	0.19±0.04	34.45±0.86	1.14±0.09	0.91±0.26	1.31±0.28	0.59±0.01	3.49±0.65	0.43±0.08

Table S4. Nucleotide sequences for primers used in the assessment of wheat gene expression, their functional importance and reference.

Gene	Primer sequence	Functional annotation	Reference
DREB2	DREB2-F: 5′-CGGAGATGCAGCTTCTTGATT-3′ DREB2-R: 5′-GATCTCGAGCG ACGGGTACTT-3′	Encoding for an abiotic stress-responsive transcription factor: dehydration responsive element binding protein 2 in wheat	[1]
CTR1	CTR1-F: 5′ -GCTGCTCTTGTTGAATCCTGTTG-3′ CTR1-R: 5′-ATCCACAATGCTTGAAAACGAA-3′	Encoding for a regulatory component of the ethylene signalling pathway that modulates stress related changes in plants	[1]
WKY26	TaWRKY26-F 5′-TCTTTGGCTTCTCCTTTCACG-3′ TaWRKY26-R 5′-TGTTGCTCACTTCTACCACTTG-3′	WRKY transcription factor 26 WRKY TFs play the important roles in regulation of water/drought-stress by modulating the cellular osmotic balance, ROS scavenging mechanism and expression of different stress-related genes [2] AtWRKY26 changed during stress treatments including thermal factors, NaCl, abscisic acid (ABA) and osmotic stress, and significantly under NaCl and cold treatments [3].	[4]
WKY71	TaWRKY71-F 5′-AAACCCGTCATCTCCAAGC-3′ TaWRKY71-R 5′-TTGTCCTTGGTCACCTTCTG-3′	TaWRKY71 gene might play important roles in seed germination and abiotic stress response (tolerant to salt and drought stresses) [5].	[4]
POD	Forward 5'-CAGCGACCTGCCAGGCTTTA-3' Reverse 5'-GTTGGCCCGGAGAGATGTGG-3'	Peroxidases in plant (PODs) are involved in various physiological functions such as removal of hydrogen peroxides, senescence, oxidation of toxic reductants, lignin formation, pathogen defense, or insect attack [6]	[7]
CAT1	Forward 5'-CCATGAGATCAAGGCCATCT-3' Reverse 5'-ATCTTACATGCTCGGCTTGG-3'	Encoding for catalase, an enzyme essential for elimination of H_2O_2 produced through photorespiration under stress conditions	[7]
LPX	TaLPX-F 5'-GAGGTTTTCAAGCGGTTCAG-3′ TaLPX-R 5'-TTGTGGTCGGAGGTGTTG-3′	Lipoxygenase Lipoxygenases (Loxs) are non-heme iron-containing dioxygenases that catalyse the oxidization of polyunsaturated fatty acids (PUFA) containing a (1Z, 4Z)-pentadiene to hydroperoxide fatty acids. The metabolites of Loxs are collectively called oxylipins, which play an important role in the regulation of plant germination, growth and development, senescence, wounding and stress responses, and pathogen defense [8]	[4]

ARF2	TaARF2-F 5′-TTAAGGTGCGTTGGGATGAG-3′ TaARF2-R 5′-TTGGCACGAGAAAGAGGAAG-3′	Auxin response factor Auxin response factors (ARFs) are plant-specific transcription factors that bind explicitly to Auxin Response Elements and mediate downstream expression of auxin responsive genes involved at various stages of plant growth and development.	[4]
CKX10	TaCKX10-F 5′-GCCATTTCAGTTTCCACGAC-3′ TaCKX10-R 5′-TCAAGAACACATGCCTCACG-3′	Cytokinin dehydrogenase Cytokinin oxidase/dehydrogenase (CKX) catalyzes irreversible degradation/ This step of CK metabolism plays an important role in the regulation of cytokinin level and regulation of yield-related traits in cereals. According to the last assignments, TaCKX10 are mainly expressed in seedling roots [9]	[4]
MAPK	TaMAPK-F 5′-CCTACTGGGTCGTTTACTTGC-3′ TaMAPK-R 5′-CGAAATTGGATGCCTTGATGG-3′	MAPK cascades are important for plant signal transduction, as they are involved in signaling of hormones, growth factors, microbes, or damage-associated molecular patterns, and they convert extracellular stimuli into intracellular responses while amplifying the transmitting signal [10]	[4]
ABARE	ABARE (F): TTACACCGTGGAGCTTGAAG ABARE (R): TTCACGTTCTCCTTGGACTG	ABA-signalling cascade	[11]
Actin	TaActin2-F 5′-TGCCCATTTACGAAGGATACG-3′ TaActin2-R 5′-GTGTTGGGTTCACAATGTCG-3′	Constitutive gen as endogenous contol.	[4]

[1] D. Barnawal, N. Bharti, S. S. Pandey, A. Pandey, C. S. Chanotiya, and A. Kalra, "Plant growth-promoting rhizobacteria enhance wheat salt and drought stress tolerance by altering endogenous phytohormone levels and TaCTR1/TaDREB2 expression," *Physiol. Plant.*, vol. 161, no. 4, pp. 502–514, Dec. 2017, doi: 10.1111/PPL.12614.

[2] D. L. Rushton *et al.*, "WRKY transcription factors: key components in abscisic acid signalling," *Plant Biotechnol. J.*, vol. 10, no. 1, pp. 2–11, Jan. 2012, doi: 10.1111/J.1467-7652.2011.00634.X.

[3] Q. T. Fu and D. Q. Yu, "[Expression profiles of AtWRKY25, AtWRKY26 and AtWRKY33 under abiotic stresses.].," *Yi Chuan*, vol. 32, no. 8, pp. 848–856, 2010, doi: 10.3724/SP.J.1005.2010.00848.

[4] X. Wu *et al.*, "Bacillus halotolerans KKD1 induces physiological, metabolic and molecular reprogramming in wheat under saline condition," *Front. Plant Sci.*, vol. 13, Aug. 2022, doi: 10.3389/FPLS.2022.978066/FULL.

[5] Q. Xu, W. J. Feng, H. R. Peng, Z. F. Ni, and Q. X. Sun, "TaWRKY71, a WRKY transcription factor from wheat, enhances tolerance to abiotic stress in transgenic Arabidopsis thaliana," *Cereal Res. Commun.*, vol. 42, no. 1, pp. 47–57, Mar. 2014, doi: 10.1556/CRC.2013.0051/METRICS.

[6] K. Yoshida, P. Kaothien, T. Matsui, A. Kawaoka, and A. Shinmyo, "Molecular biology and application of plant peroxidase genes," *Appl. Microbiol. Biotechnol.*, vol. 60, no. 6, pp. 665–670, 2003, doi: 10.1007/S00253-002-1157-7/METRICS.

[7] N. Bharti, S. S. Pandey, D. Barnawal, V. K. Patel, and A. Kalra, "Plant growth promoting rhizobacteria Dietzia natronolimnaea modulates the expression of stress responsive genes providing protection of wheat from salinity stress," *Sci. Reports 2016 61*, vol. 6, no. 1, pp. 1–16, Oct. 2016, doi: 10.1038/srep34768.

[8] P. Veronico *et al.*, "A novel lipoxygenase in pea roots. Its function in wounding and biotic stress," *Plant Physiol.*, vol. 141, no. 3, pp. 1045–1055, 2006, doi: 10.1104/PP.106.081679.

[9] B. Jablonski *et al.*, "Tackx2.2 genes coordinate expression of other tackx family members, regulate phytohormone content and yield-related traits of wheat," *Int. J. Mol. Sci.*, vol. 22, no. 8, p. 4142, Apr. 2021, doi: 10.3390/IJMS22084142/S1.

[10] X. Liu *et al.*, "MAPK-mediated auxin signal transduction pathways regulate the malic acid secretion under aluminum stress in wheat (Triticum aestivum L.)," *Sci. Reports 2017 71*, vol. 7, no. 1, pp. 1–12, May 2017, doi: 10.1038/s41598-017-01803-3.

[11] M. Ayaz *et al.*, "Salt Tolerant Bacillus Strains Improve Plant Growth Traits and Regulation of Phytohormones in Wheat under Salinity Stress," *Plants*, vol. 11, no. 20, p. 2769, Oct. 2022, doi: 10.3390/PLANTS11202769/S1.
